# Renal effects of selective cyclooxygenase-2 inhibitor anti-inflammatory drugs: A systematic review and meta-analysis

**DOI:** 10.1016/j.rcsop.2024.100475

**Published:** 2024-07-08

**Authors:** Tayanny Margarida Menezes Almeida Biase, João Gabriel Mendes Rocha, Marcus Tolentino Silva, Inês Ribeiro-Vaz, Taís Freire Galvão

**Affiliations:** a School of Pharmaceutical Sciences, Universidade Estadual de Campinas, Campinas, São Paulo, Brazil; b Department of Public Health, School of Health Sciences, University of Brasília, Brasília, Brazil; c Department of Community Medicine, Health Information and Decision, School of Medicine, University of Porto, Porto, Portugal; d Center for Health Technology and Services Research, School of Medicine, University of Porto, Porto, Portugal

**Keywords:** Cyclooxygenase-2 inhibitors, Adverse events, Edema, Hypertension, Renal, Systematic review

## Abstract

**Background:**

Selective cyclooxygenase-2 inhibitor anti-inflammatory drugs (coxibs) are associated with the development of adverse events, mainly gastrointestinal and cardiovascular, but renal effects are less known.

**Objective:**

To assess the renal risks of coxibs compared to placebo by means of a systematic review and meta-analysis.

**Methods:**

Randomized controlled trials that assessed renal effects of coxibs (celecoxib, etoricoxib, lumiracoxib, parecoxib, and valdecoxib) were searched in PubMed, Embase, Scopus and other sources up to March 2024. Two independent reviewers performed study screening, data extraction, and risk of bias assessment. Random effect meta-analysis was employed to calculate the relative risks (RR) and 95% confidence intervals (CI) of renal effects of coxibs compared to placebo and inconsistency among studies (*I*^*2*^). Certainty of evidence was assessed using the Grading of Recommendations Assessment, Development and Evaluation approach.

**Results:**

Out of 5284 retrieved records, 49 studies (comprising 46 reports) were included. Coxibs increased the risk of edema (RR 1.46; 95% CI 1.15, 1.86; *I*^*2*^ = 0%; 34 studies, 19,754 participants; moderate-certainty evidence), and celecoxib increased hypertensive or renal events (RR 1.24; 95% CI 1.08, 1.43; *I*^*2*^ = 0%; 2 studies, 3589 participants; moderate-certainty evidence). Etoricoxib increased the risk of hypertension (RR 1.98; 95% CI 1.14, 3.46; *I*^*2*^ = 34%; 13 studies, 6560 participants; moderate-certainty evidence); no difference was observed when pooling all coxibs (RR 1.26; 95% CI 0.91, 1.76; *I*^*2*^ = 26%; 30 studies, 16,173 participants; moderate-certainty evidence).

**Conclusions:**

Coxibs likely increase the renal adverse effects, including hypertension and edema. Awareness about the renal risks of coxibs should be increased, mainly in high-risk patient.

## Highlights

• Selective cyclooxygenase-2 inhibitor anti-inflammatory drugs (coxibs) increases renal adverse effects.
• Moderate certainty of evidence indicates all coxibs increased the risk of edema.
• Etoricoxib increased the incidence of hypertension, with moderate certainty.
• Raise the awareness of coxibs' renal risks is needed, mainly for high renal risk groups.

## 1 Introduction

Selective cyclooxygenase-2 inhibitor anti-inflammatory drugs (coxibs) are a specific category of nonsteroidal anti-inflammatory medications developed to reduce the production of inflammatory substances while preserving the integrity of the gastric lining, such a reduced risk of gastric and duodenal ulcers. They are prescribed to alleviate symptoms of inflammation and acute pain.^1^^,^^2^

Celecoxib and rofecoxib were the first coxibs approved by the US Food and Drug Administration in the late 1990's.^3^^,^^4^ After its introduction on the market, coxibs showed important cardiovascular, renal, and gastrointestinal adverse events,^5^ leading to the withdrawal of rofecoxib from commercialization due to its increased cardiovascular effects (including stroke and death), concerns regarding the safety of coxibs intensified.^6^ This major event led health authorities and experts to question the safety of other available coxibs,^7^ issuing warnings to healthcare professionals and the general public regarding the use of several coxibs.^8^

Other adverse effects from the short- or long-term uses of coxibs, such as renal effects, are less disseminated for both patients and clinicians. For instance, few systematic reviews have examined the renal safety of coxibs. Available syntheses are mostly based on observational studies, yielding inconclusive results regarding the statistical significance of the association between coxib use and the risk of renal damage.^9^^,^^10^ The systematic review based on randomized clinical trials was conducted in 2006, indicating a higher risk of peripheral edema, hypertension and renal dysfunction associated with the use of rofecoxib – the coxib withdrawn from market in early 2000's – and no assessment of quality of evidence was provided.^11^

The kidneys are responsible for filtering the blood and regulating the balance of fluids and electrolytes in the body.^12^ Hypertension is associated with the activation of the renin-angiotensin-aldosterone system, leading to sodium and water retention, which contributes to increased blood volume and blood pressure.^12^ When renal dysfunction is compromised, often exacerbated by hypertension, it can result in fluid and electrolyte retention in tissues, manifesting as edema. These processes underscore the importance of renal function in hemodynamic and electrolyte regulation, directly influencing the clinical presentation and therapeutic management of conditions such as hypertension and edema.^13^

Taking all reasons above and the lack of updated synthesis along with certainty of evidence about the renal risks from the use of coxibs, present study aimed to assess the renal effects of coxibs by means of a systematic review with meta-analysis.

## 2 Methods

### 2.1 Protocol and registration

The protocol of this systematic review and meta-analysis was previously registered in the International prospective register of systematic reviews database (PROSPERO, registration number: CRD42022380227). The findings were reported according to the Preferred Reporting Items for Systematic Reviews and Meta-Analyses (PRISMA) guidelines.^14^

### 2.2 Eligibility criteria

Randomized controlled trials that assessed the renal effects of coxibs (celecoxib, etoricoxib, lumiracoxib, parecoxib, and valdecoxib) compared to a placebo were considered as eligible studies. Clinical trials that assessed more than one coxib as an intervention and compared them with placebo were also eligible provided that the results were described separately. In PICOS format, the research question was structured as follows:

• **P**articipants: adults
• **I**ntervention: coxibs
• **C**ontrol: placebo
• **O**utcome: renal adverse events
• **S**tudy type: randomized controlled trials

Studies that only assessed rofecoxib were excluded due to its withdrawal from the market in 2004. No restriction was set concerning the year of publication or the language of the study. All efforts were made to obtain the full text of eligible studies for inclusion in this review.

### 2.3 Data sources and search strategy

Studies were sought in PubMed, Embase, Central, Scopus, and Web of Science bibliographic databases. Following the Peer Review of Electronic Search Strategies (PRESS) guideline,^15^ a pilot strategy for PubMed was developed and revised by a researcher with experience in systematic reviews. The final search strategy was adapted for the other databases. Email alerts have been configured to identify new potentially eligible publications and thus plan the search update. All strategies are available at Supplementary Material 1. The searches were carried out up to September 2022 and fully updated in March 2024. The files were imported to Covidence (www.covidence.org) for study selection, extraction, and risk of bias assessment.^16^

### 2.4 Selection process

Initially, 100 studies were screened by the two researchers responsible for study selection (TMB, JGMR) as a piloting step. Disagreements were discussed in calibration meetings to consolidate the inclusion criteria. After this procedure, study eligibility was independently assessed by the two researchers by screening titles and abstracts and disagreements were solved by consensus or by a third researcher (TFG). The same process was performed for full text assessment of the eligible studies screened.

### 2.5 Data collection process and data items

A form was created on Covidence (www.covidence.org) to extract data based on the variables of interest.^16^ A pilot extraction of two studies was performed to discuss disagreements and adjust the calibration meeting data collection form. Data were extracted independently by the two researchers involved in the selection process and disagreements were resolved by a third researcher. The following data were extracted from the studies: study, study dates, location, clinical condition of participants, population, coxib treatment(s), dose, number of participants in the coxib group, number of participants in the placebo group, trial duration, renal effects (elevated creatinine, renal or hypertensive, renal and urinary disorders, elevated blood urea nitrogen, hypertension, edema, glycosuria, hematuria, urinary retention, urinary tract infection, and discontinuation due to elevated creatinine, hypertension, and edema), and sponsorship source. The composite outcome ‘renal or hypertensive’, was a combination of two or more of the following: elevated creatinine, fluid retention, edema, hypertension, proteinuria, and renal failure. As for renal and urinary disorders, hematuria, nephrolithiasis, nephroptosis, nephrotic syndrome, acute and chronic renal failure, urethral stricture, urinary incontinence, and urinary retention were considered.

### 2.6 Study risk of bias assessment

Risk of bias assessment was independently conducted by two authors (TMB, JGMR) and confirmed by a third author (TFG) using the Cochrane Collaboration tool for risk of bias assessment (RoB 1.0).^17^ The following characteristics were evaluated: (1) sequence generation of the allocation sequence; (2) allocation concealment of the patient group; (3) blinding of participants and personnel from knowledge of which intervention a participant received; (4) blinding of outcome assessment; (5) incomplete outcome data; (6) selective reporting; and (7) other sources of bias, which accounted for important concerns unaddressed in another domain. Each domain was classified as either ‘high’ or ‘low’ risk of bias. Disagreements were resolved by discussions between the two reviewers during a consensus meeting and, when necessary, with the participation of another member of the review team to make a final decision (TFG).

### 2.7 Data analysis

The relative risk (RR), with 95% confidence interval (CI 95%), of renal effects of coxibs compared to placebo was calculated by meta-analysis using the DerSimonian and Laird method and random effect models, assuming heterogeneity between studies. Subgroup analyses by type of coxib were conducted to investigate pertinent specificities. Heterogeneity between studies was analyzed by the chi-squared test, adopting a significance level of *p* < 0.10 and the magnitude of inconsistency was estimated by I-squared statistics (*I*^*2*^). The direction of the result of each study was weighted, in which the I^2^ and the result of the chi-square test to classify the heterogeneity into not important, moderate, substantial, or considerable.^18^ In heterogeneous outcomes with >10 studies included, meta-regressions were carried using the test by Knapp and Hartung^19^ to identify the effect of independent variables (decade, age of participants and risk of bias of studies) in the outcome variability. All analyses were calculated on Stata Version 14.2 (College Station, TX) using the *metan* command.

### 2.8 Reporting bias assessment

For outcomes with at least 10 studies, small-study effects (publication bias) was assessed by visually inspection of the funnel plots' asymmetry and by calculating Harbord's modified test for small-study effects (*p* < 0.10). Selective outcome reporting was assumed in studies that either (i) adopted an incidence threshold of adverse effects to report them, or (ii) reported only pre-specified or previously defined adverse events, without reporting all adverse events that occurred.

### 2.9 Certainty assessment

The outcomes importance was classified based on a scale from 1 to 9 into critical (9–6), important (6–4) or of limited importance (3–1) for decision-making,^20^ considering the probability of the outcome to incur in renal impairment, aligned to a guideline used to detect kidney injuries^21^ and the possibility of a coxib to cause this outcome. In this sense, altered creatinine was classified as critical because it can be an important biomarker of renal impairment and it could be caused by coxibs, whereas renal infection was classified as not important despite its possible severity as the chances a drug causing it are very low, aligned to a guideline used to detect kidney injuries.^21^ The information was validated by an experienced nephrologist during a meeting. Thus, the following classification of importance of outcomes: elevated creatinine (score 9), renal or hypertensive (9), renal and urinary disorders (9), elevated blood urea nitrogen (9) and hypertension (7) as critical; edema (4), glycosuria (4), hematuria (4) as important, and urinary retention (3), urinary tract infection (1), and discontinuation due to elevated creatinine, hypertension, and edema (1) as of limited importance. Each outcome was measured by the Grading of Recommendations, Assessment, Development, and Evaluations method (GRADE) to rate certainty of evidence as high, moderate, low or very low, based on the assessment of the risk of bias, indirect evidence, inconsistency, imprecision, and publication bias of each outcome.^20^ The GRADE profiler Guideline Development Tool (GRADEpro GDT) was used to elaborate the summary of findings table (https://gradepro.org).^22^

## 3 Results

### 3.1 Study selection

**Fig. 1:**
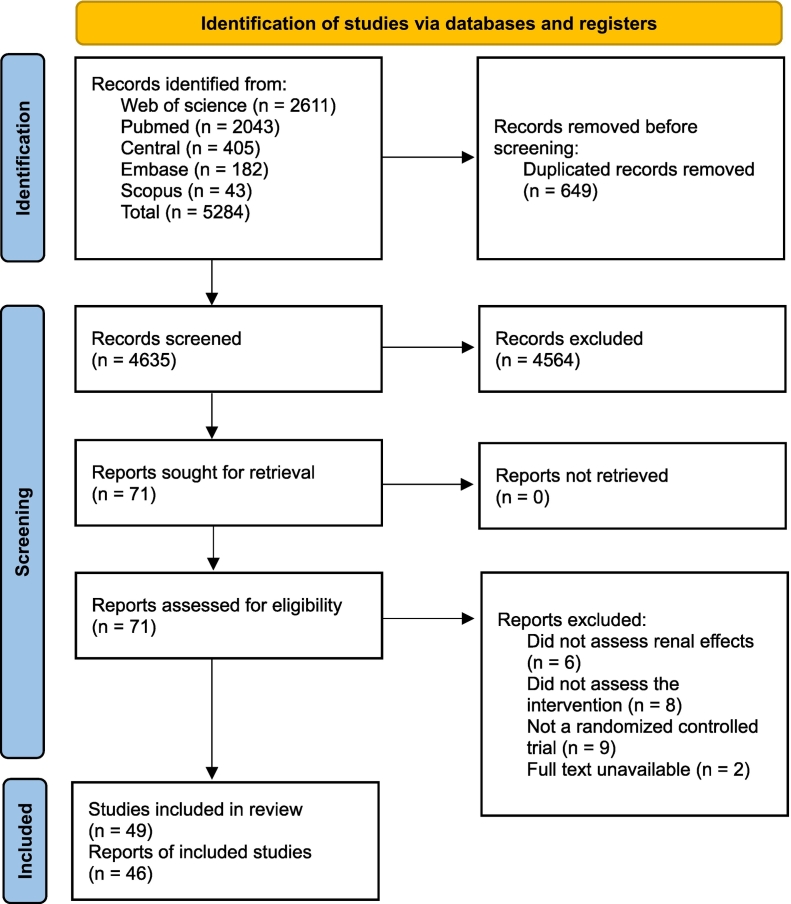
Process of search, selection, and inclusion of studies.

In total, 5284 records were identified in the selected databases. After removing duplicates, 4635 records were assessed for inclusion, of which 71 were screened in full against the inclusion criteria and 49 studies were included, which actually comprised 46 reports,^23^, ^24^, ^25^, ^26^, ^27^, ^28^, ^29^, ^30^, ^31^, ^32^, ^33^, ^34^, ^35^, ^36^, ^37^, ^38^, ^39^, ^40^, ^41^, ^42^, ^43^, ^44^, ^45^, ^46^, ^47^, ^48^, ^49^, ^50^, ^51^, ^52^, ^53^, ^54^, ^55^, ^56^, ^57^, ^58^, ^59^, ^60^, ^61^, ^62^, ^63^, ^64^, ^65^, ^66^, ^67^, ^68^ as three articles presented results from two studies each (Fig. 1). Supplementary Material 2 lists the references of excluded studies according to the reason for exclusion.

### 3.2 Study characteristics

Supplementary Material 3 shows the characteristics of the studies included in the analysis. In total, 20,337 participants were included in the studies, which were conducted between 1999 and 2014, and primarily involved adults, with follow-up durations ranging from 1 day to 3 years. The most frequent clinical conditions reported in the studies were osteoarthritis (*n* = 21) and rheumatoid arthritis (*n* = 8). Most studies assessed celecoxib (*n* = 25), etoricoxib (*n* = 17), and lumiracoxib (n = 8). Most studies received funding from a pharmaceutical industry that manufactured coxibs reported at the time studies were published, of which the main sources of funding refer to Merck (*n* = 19) and Novartis (n = 8).

### 3.3 Risk of bias in studies

**Fig. 2:**
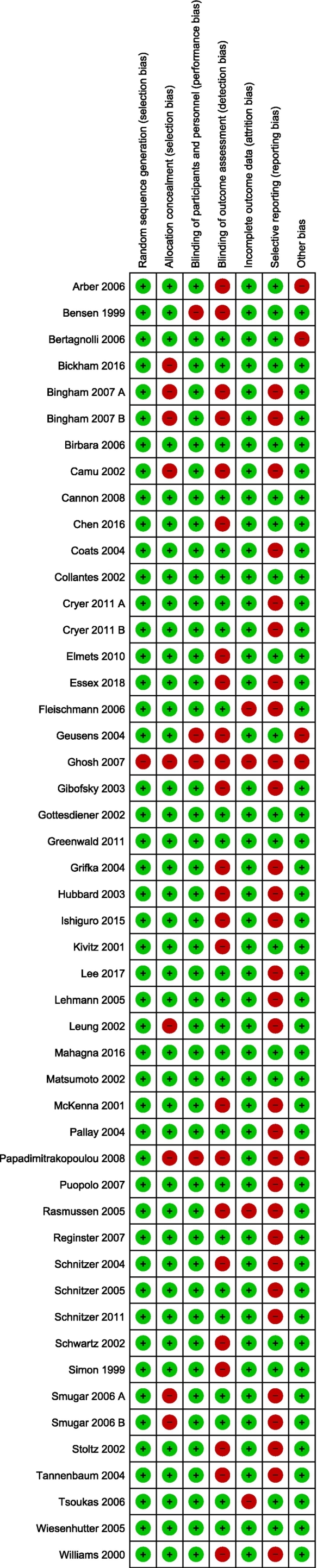
Risk of bias of the included studies.

Fig. 2 summarizes the assessed risk of bias for each study in this meta-analysis. Most studies showed a high risk of bias due to selective outcome reporting (*n* = 30), lack of outcome assessment blinding (*n* = 24), and insufficient information regarding allocation concealment (*n* = 9).

### 3.4 Results of syntheses

Coxibs did not significantly affect creatinine levels when compared with placebo (RR 0.73; 95% CI 0.21, 2.55; *I*^*2*^ = 38%; 4 studies, 3195 participants; low-certainty evidence). This was also observed in the celecoxib, lumiracoxib, and valdecoxib subgroups (Table 1).

**Table 1:** Summary of findings of critical and important outcomes for decision-making and certainty of evidence of the renal effects of selective cyclooxygenase-2 inhibitor anti-inflammatory drugs compared to placebo, in relative risk (RR) and 95% confidence interval (95%CI).

Outcomes	N° of participants (studies)	Certainty of the evidence	RR (95%CI)	Anticipated absolute effects	
				Risk in placebo	Risk difference with coxib
Critical outcomes					
Elevated creatinine	3195 (4)	⨁⨁◯◯ Low^a^, ^b^, ^c^	0.73 (0.21, 2.55)	11 per 1000	3 fewer per 1000 (9 fewer to 17 more)
Renal or hypertensive outcomes (celecoxib)	3589 (2)	⨁⨁⨁◯ Moderate^d^	1.24 (1.08, 1.43)	173 per 1000	42 more per 1000 (12 more to 75 more)
Renal and urinary disorders (celecoxib)	4244 (3)	⨁⨁◯◯ Low^b^, ^d^	1.18 (0.65, 2.15)	11 per 1000	2 more per 1000 (4 fewer to 12 more)
Elevated blood urea nitrogen (BUN)	1282 (3)	⨁⨁⨁◯ Moderate^b^, ^c^	2.21 (0.35, 13.95)	0 per 1000	0 fewer per 1000 (0 fewer to 0 fewer)
Hypertension	16,173 (30)	⨁⨁⨁◯ Moderate^b^	1.26 (0.91, 1.76)	17 per 1000	5 more per 1000 (2 fewer to 13 more)
Important outcomes					
Edema	19,754 (34)	⨁⨁⨁◯ Moderate^e^	1.46 (1.15, 1.86)	16 per 1000	7 more per 1000 (2 more to 14 more)
Hematuria	1133 (2)	⨁⨁⨁◯ Moderate^b^, ^c^	1.81 (0.60, 5.43)	14 per 1000	11 more per 1000 (6 fewer to 62 more)

a High risk for incomplete outcome data.

b Confidence interval not significant.

c Many studies reporting this outcome used a threshold to report adverse reactions.

d Most studies were stopped earlier than planned.

e Most studies were not blinded for outcome assessment.

The risk of renal or hypertensive events was found to increase with celecoxib compared to placebo (RR 1.24; 95% CI 1.08, 1.43; *I*^*2*^ = 0%; 2 studies, 3589 participants; moderate-certainty evidence).

Renal and urinary disorders did not differ significantly (RR 1.18; 95% CI 0.65, 2.15; *I*^*2*^ = 0%; 3 studies, 4244 participants; low-certainty evidence), including blood urea nitrogen levels (RR 2.21; 95% CI 0.35, 13.95; *I*^*2*^ = 0%; 3 studies, 1282 participants; moderate-certainty evidence) and its subgroups (celecoxib, etoricoxib, and valdecoxib).

**Fig. 3:**
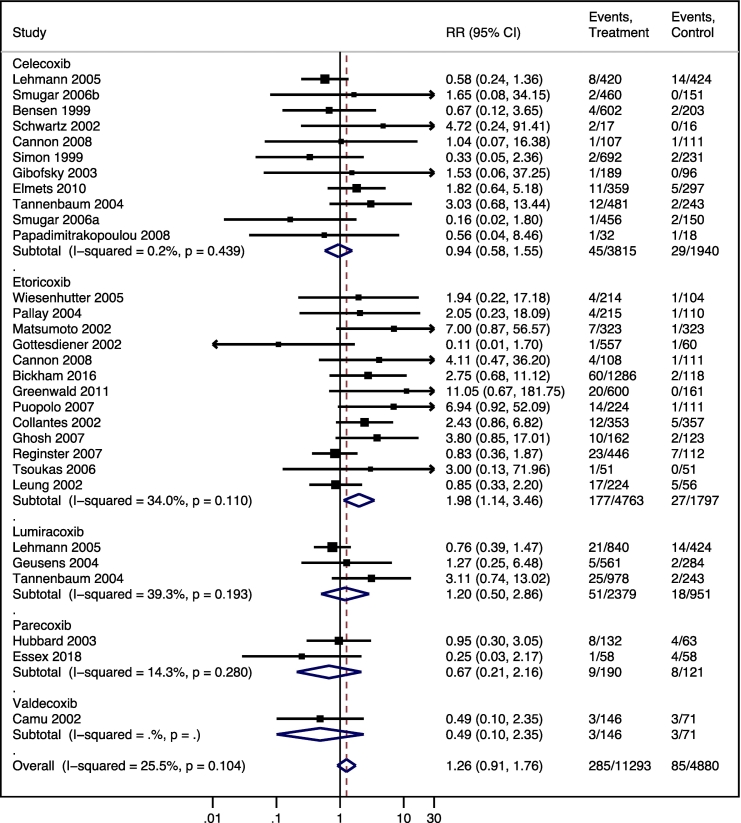
Relative risk (RR) and 95% confidence interval (CI) of hypertension for selective cyclooxygenase-2 inhibitor anti-inflammatory drugs compared to placebo.

Etoricoxib was associated with an increased risk of hypertension (RR 1.98; 95% CI 1.14, 3.46; *I*^*2*^ = 34%; 13 studies, 6560 participants; moderate-certainty evidence), while all coxibs (RR 1.26; 95% CI 0.91, 1.76; *I*^*2*^ = 26%; 30 studies, 16,173 participants; moderate-certainty evidence), including celecoxib, lumiracoxib, parecoxib, and valdecoxib subgroups, did not affect this outcome (Fig. 3). Meta-regressions of this outcome by decade (*p* = 0.992; Supplementary Material 4), age of participants (*p* = 0.514; Supplementary Material 5), and studies' risk of bias (*p* = 0.849; Supplementary Material 6) did not identify sources of heterogeneity considering all coxibs.

**Fig. 4:**
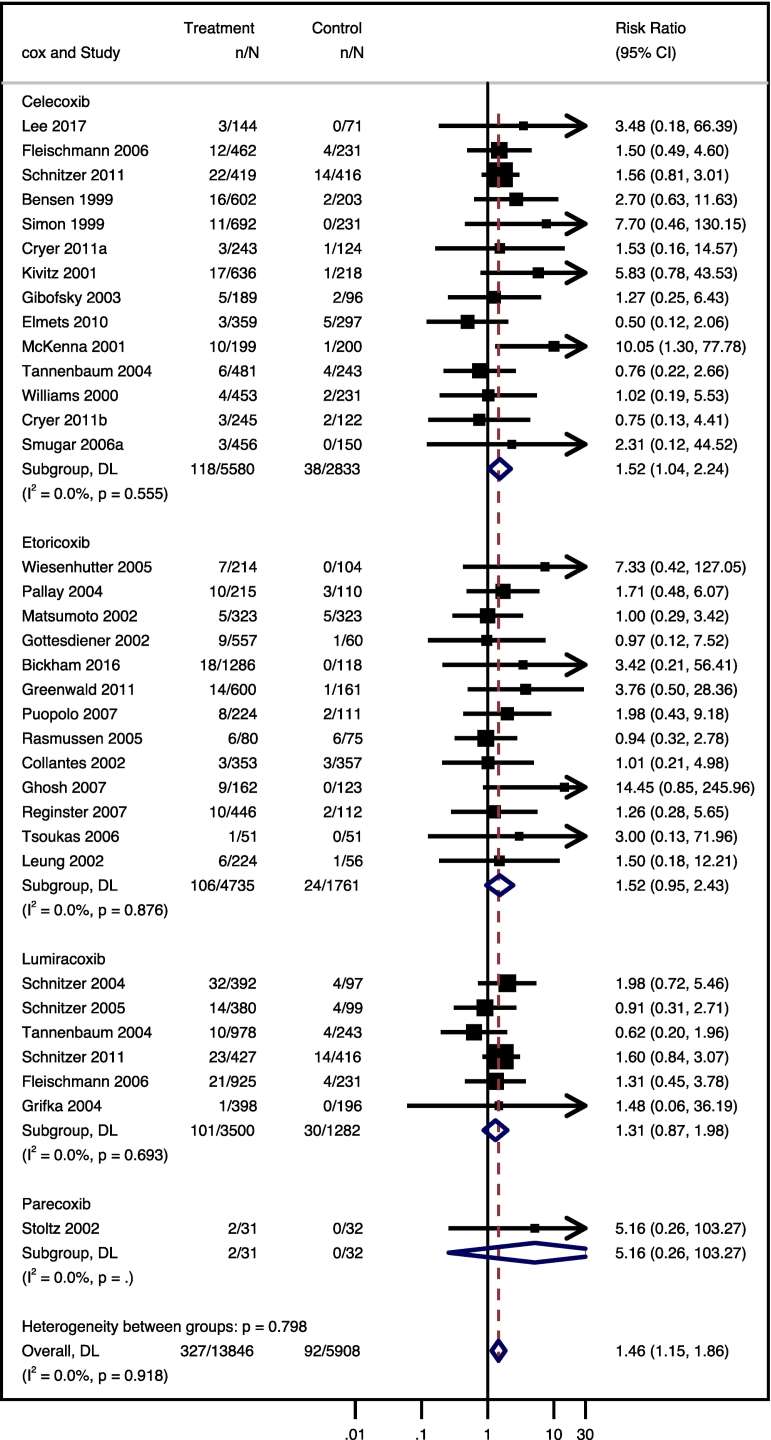
Relative risk (RR) and 95% confidence interval (CI) of edema for selective cyclooxygenase-2 inhibitor anti-inflammatory drugs compared to placebo.

Coxibs increased the risk of edema compared to placebo (RR 1.46; 95% CI 1.15, 1.86; *I*^*2*^ = 0%; 34 studies, 19,754 participants; moderate-certainty evidence). The celecoxib subgroup also increased the risk (RR 1.52; 95% CI 1.04, 2.24; *I*^*2*^ = 0%; 14 studies, 8413 participants; moderate-certainty evidence), while etoricoxib, lumiracoxib, and parecoxib did not significantly affect this outcome (Fig. 4). As the outcome was homogeneous, despite including over 10 studies, meta-regressions were not necessary.

Glycosuria (RR 0.40; 95% CI 0.11, 1.46; 1 study, 372 participants; moderate-certainty evidence), hematuria (RR 1.81; 95% CI 0.60, 5.43; *I*^*2*^ = 0%; 2 studies, 1133 participants; moderate-certainty evidence), and urinary retention (RR 0.96; 95% CI 0.13, 7.15; *I*^*2*^ = 57%; 3 studies, 487 participants; very low-certainty evidence) were not impacted by coxib use.

The risk of urinary tract infection also showed no differences (RR 0.81; 95% CI 0.56, 1.16; *I*^*2*^ = 0%; 10 studies, 6587 participants; low-certainty evidence), including the celecoxib, etoricoxib, lumiracoxib, and valdecoxib subgroups.

Discontinuation due to elevated creatinine (RR 0.75; 95% CI 0.03, 18.30; 1 study, 1221 participants; moderate-certainty of evidence), hypertension (RR 0.99; 95% CI 0.46, 2.15; *I*^*2*^ = 0%; 10 studies, 5769 participants; low-certainty of evidence), and edema (RR 1.75; 95% CI 0.77, 3.98; *I*^*2*^ = 0%; 13 studies, 7109 participants; moderate-certainty of evidence) were not significant, including their subgroups of coxibs.

### 3.5 Reporting biases

Publication bias was not suspected for edema (*p* = 0.381) and hypertension (*p* = 0.743) in the comparisons between celecoxib and etoricoxib, as the distribution of the effects by the study size was symmetric (Supplementary Material 7 and 8).

### 3.6 Certainty of evidence

Table 1 summarizes critical and important outcomes for decision-making and certainty of evidence. Other outcomes are presented in Supplementary Material 9. Urinary retention outcome was rated as very low certainty of evidence due to the imprecision of estimates, risk of bias, imprecision, and inconsistency. Elevated creatinine, renal and urinary disorders, urinary tract infection, and discontinuation due to hypertension had a low certainty of evidence due to risk of bias and imprecision. Renal or hypertensive outcomes, elevated blood urea nitrogen, hypertension, edema, glycosuria, hematuria, and discontinuation due to elevated creatinine and edema had moderate certainty of evidence due to risk of bias or imprecision.

## 4 Discussion

Coxibs significantly increased the risk of adverse renal events compared to placebo, especially edema and renal or hypertensive events. The certainty of the evidence for these results ranged from low to moderate. Etoricoxib increased the risk of hypertension and celecoxib, the risk of edema and renal or hypertensive events. No differences were observed for elevated creatinine, renal and urinary disorders, increased blood urea nitrogen, glycosuria, hematuria, urinary retention, urinary tract infection, and discontinuation due to elevated creatinine, hypertension, and edema. Lumiracoxib, parecoxib, and valdecoxib were assessed in fewer clinical trials with smaller participant's number, resulting in limited data on renal risk-related outcomes of interest. This limitation in the number of studies and data may contribute to imprecisions in comparisons.

Eligible studies included clinical trials that mostly aimed to assess the efficacy of coxibs, rather than to specifically evaluate renal adverse events. Almost half of the included studies adopted a threshold of incidence of adverse effects to report them ranging from 1 to 5%. This is an important selective reporting bias that may underestimate the risk of renal effects. Many of the included studies exhibited shortcomings, such as inadequate blinding of researchers, and lack of details on participant selection for the different treatment groups. These limitations may compromise the validity and interpretation of the meta-analysis results. Studies were published from 1999 to 2018 but those studies to 2010 accounted for more than three quarters of the included studies. At that time, the lack of guidelines hindered the clear reporting of scientific studies, leading to selective reporting practices and the omission of important information. The introduction of the Consolidated Standards of Reporting Trials (CONSORT) in 1996 aimed to improve the reporting of clinical trials, making them more transparent, reliable, and useful to the scientific community and medical practice.^69^ Adherence to reporting guidelines potentially improves the quality and integrity of the research, as observed in a 2010 systematic review that included 53 reports, 25 of which showed improved integrity by adopting this tool.^70^ Present study fills a gap in the literature by offering a comprehensive analysis of the renal effects of coxibs, using a rigorous methodology. By synthesizing data from a wide range of clinical studies, a more complete insight was provided into the potential renal risks associated with the use of these drugs.

Low certainty of evidence indicates that coxibs increased the risk of edema, i.e., the accumulation of fluid in tissues. This certainty suggests it is very likely that future studies will have a significant impact on confidence in the estimate of effect. Edema can affect any part of the body and show a variety of clinical signs and symptoms, which can range from localized swelling to a more generalized condition known as anasarca, characterized by extensive swelling throughout the body,^71^ resulting in visible clinical signs and symptoms. Celecoxib was also significantly associated to a high incidence of edema, with moderate certainty evidence, which indicates that future studies may alter confidence in the estimate of effect or even modify the estimate itself. This fluid buildup may stem from increased blood volume, which can contribute to hypertension, especially when kidney factors are involved.^72^ Elevated blood pressure, in turn, can increase fluid leakage into tissues, leading to the development of edema.^72^ The risks associated with edema are related to possible complications, such as secondary infections, skin ulcers, mobility difficulties, and circulatory disorders.^73^ Additionally, edema can be a symptom of more serious underlying conditions, such as congestive heart failure, chronic kidney disease, or liver problems.^71^, ^72^, ^73^ The results are consistent with a 2006 meta-analysis that investigated renal adverse events associated with coxibs and found that these drugs, especially rofecoxib (which this review ignored due to its withdrawal from the market), increased the risk of peripheral edema.^11^

Celecoxib increased the risk of renal or hypertensive events, which encompass a combined of outcomes including elevated creatinine, fluid retention, edema, hypertension, and renal failure. The certainty of the evidence for this result was moderate. The pathogenesis of hypertensive renal damage may happen due to the failure of renal autoregulatory mechanisms to prevent hypertension, causing a vicious cycle.^74^ According to present findings, celecoxib use may initiate this imbalance. Renal failure may be identified by proteinuria, an important biomarker of this condition and of chronic kidney disease.^75^ These conditions may also lead to edema, as discussed before. The findings are in agreement with a 2009 meta-analysis, which showed that coxibs may increase the risk of cardiovascular events such as hypertension and edema^76^ and trigger a cycle of renal or hypertensive events.

Low certainty of evidence indicates that etoricoxib increased this risk of hypertension, which was insignificant for other coxibs. These results agree with a 2017 systematic review that assessed the safety of coxibs in patients with osteoarthritis and found that the use of celecoxib, rofecoxib, and etoricoxib increased the risk of hypertension.^76^ Hypertension is a major risk factor for cardiovascular disease and overall mortality levels.^12^ It is strongly related to chronic kidney disease since hypertension can damage the kidneys and the progression of the chronic kidney disease can worsen hypertension.^12^ In chronic kidney disease, the pathophysiology of hypertension is complex and involves several factors, such as a decrease in functional nephrons, sodium retention, increased blood volume, upregulation of the sympathetic nervous system and of the renin-angiotensin-aldosterone system, and endothelial dysfunction.^12^^,^^77^ The inadequate control of hypertension can accelerate its progression to end-stage kidney disease, increasing the risk of kidney complications such as nephrolithiasis and urinary tract infections. In severe cases, untreated hypertension can result in kidney failure, requiring dialysis or a kidney transplant.^78^

The pooled analysis showed that coxibs failed to affect creatinine, urea nitrogen, glycosuria, and hematuria levels and urinary retention. These indicators may signal kidney damage or dysfunction. Creatinine and nitrogenous urea are metabolic residues that, when elevated in the blood, may indicate decreased kidney function.^79^^,^^80^ Glycosuria may indicate poor diabetes or metabolic disorders control.^81^ Hematuria refers to blood in the urine, suggesting injury or inflammation in the kidneys or urinary tract.^82^ Urinary retention can pressure the kidneys and lead to progressive damage, which may stem from obstructions, bladder dysfunction, or other conditions that affect the normal flow of urine.^83^

Coxib use also failed to affect discontinuation due to elevated creatinine, hypertension and edema. No evidence related to coxib discontinuation due to renal outcomes was found in the literature. Several reasons can lead patients to discontinue treatment: adverse reactions (including pharmacological factors), individual tolerance, and clinical impacts. Some patients may be more sensitive or susceptible to certain side effects, even at therapeutic doses. These adverse reactions can range from symptoms to gastrointestinal disturbances to more severe allergic reactions.^84^

Coxibs failed to affect urinary tract infections, with very low certainty evidence. This certainty of evidence indicates that any estimate of effect should be viewed as uncertain. Leukocytes in urine may indicate an infection in the urinary tract (pyelonephritis^85^) or inflammation in the kidneys (nephritis^86^). Both pyelonephritis and nephritis can pose significant risks to the kidneys. Untreated or recurrent pyelonephritis can lead to permanent kidney tissue damage, scarring, and kidney dysfunction.^85^ Nephritis can affect the function of the glomeruli, leading to problems with kidney filtration and excretion and contributing to the development of chronic kidney disease.^86^

Based on the results of present study, several measures to guide future clinical practice can be considered. Coxibs, especially celecoxib and etoricoxib, were associated with a higher risk of adverse renal events, such as edema and hypertension. When prescribing medications for patients with pain or inflammation, healthcare professionals should consider the renal safety profile of coxibs and evaluate the potential risks and benefits for each patient. Given this association, it may be necessary to establish monitoring protocols for patients undergoing treatment with coxibs. This could include regular monitoring of blood pressure, serum creatinine, and signs of edema during treatment. These measures will contribute to a safer and more informed clinical practice in the management of pain and inflammation.

## 5 Conclusion

Coxibs increased the incidence of renal effects such as edema and renal or hypertensive outcomes. Celecoxib increased the risk of edema and renal or hypertensive outcomes and etoricoxib increased the risk of hypertension. The certainty of this evidence was moderate, and further research may impact these effects. Patients and clinicians should be aware of the renal risks of using these drugs, even in the short-term. It is important to closely monitor patients at high renal risk to prevent renal damage. Continued research is needed to improve the understanding of the risks of coxibs use, aiming for safety and improved clinical practice.

## Funding statement

The research was supported by 10.13039/501100001807São Paulo Research Foundation (FAPESP, Grant 2022/06933–8) granted to Biase TMMA. Galvao TF receives productivity scholarship from the National Council for Scientific and Technological Development (CNPq, Grant 313431/2023-0).

## Ethics in publishing

The content of the publication is the sole responsibility of the authors. The work is in accordance with editorial standards and has no plagiarism.

## Submission declaration and verification

The authors declare that article is original and has been submitted for publication in any other periodical, whether in part or in its entirety. We further declare that, once published, it will never be submitted by any of the authors to any periodical.

## Use of inclusive language

The authors declare the use of inclusive language in the publication's content.

## Author contributions

Biase TMMA, Ribeiro-Vaz I, Silva MT, Galvao TF contributed in the conception and design of the work. Biase TMMA and Rocha JB contributed for the acquisition and interpretation of data for the work and drafted the manuscript. Biase TMMA, Silva MT, Galvao TF analyzed and interpreted the data for the work. Ribeiro-Vaz I, Silva MT, Galvao TF revised the manuscript critically for important intellectual content. All authors approved the al of the version to be published and agree to be accountable for all aspects of the work in ensuring that questions related to the accuracy or integrity of any part of the work are appropriately investigated and resolved.

## CRediT authorship contribution statement

**Tayanny Margarida Menezes Almeida Biase:** Writing – review & editing, Writing – original draft, Visualization, Methodology, Investigation, Funding acquisition, Formal analysis, Data curation, Conceptualization. **João Gabriel Mendes Rocha:** Writing – original draft, Methodology, Investigation, Formal analysis, Data curation, Conceptualization. **Marcus Tolentino Silva:** Writing – review & editing, Writing – original draft, Visualization, Validation, Software, Methodology, Investigation, Formal analysis, Data curation, Conceptualization. **Inês Ribeiro-Vaz:** Writing – review & editing, Writing – original draft, Validation, Conceptualization. **Taís Freire Galvão:** Writing – review & editing, Writing – original draft, Visualization, Validation, Supervision, Methodology, Investigation, Funding acquisition, Formal analysis, Data curation, Conceptualization.

## Declaration of competing interest

The authors declare that they have no known competing financial interests or personal relationships that could have appeared to influence the work reported in this paper.

## Appendix A Supplementary data

Supplementary material: Search strategies, list of excluded studies, characteristic of included studies, and meta-regression results Image 1
