# Supplementary material for: Renal effects of selective cyclooxygenase-2 inhibitor anti-inflammatory drugs: A systematic review and meta-analysis
Source: Explor Res Clin Soc Pharm. 2024 Jul 8;15:100475. doi: 10.1016/j.rcsop.2024.100475 (PMC11304066; doi:10.1016/j.rcsop.2024.100475)
Supplement: Supplementary file 1 — Supplementary material: Search strategies, list of excluded studies, characteristic of included studies, and meta-regression results [file mmc1.docx]

**Supplementary Material 1:** Detailed search strategies in searched bibliographic databases

| Databases | Search Strategy |
| --- | --- |
| PubMed | (cox 2 inhibitor OR coxib) AND (kidney OR renal OR tolerability OR safety) AND ((randomized controlled trial[pt]) OR (controlled clinical trial[pt]) OR randomized[tiab] OR placebo[tiab] OR (drug therapy[sh]) OR randomly[tiab] OR trial[tiab] OR groups[tiab]) NOT (animals[mh] NOT humans[mh]) |
| Embase | ('cyclooxygenase 2 inhibitor'/exp OR coxib) AND ('kidney'/exp OR 'renal'/exp OR 'drug tolerability'/exp OR 'safety'/exp) AND ('randomized controlled trial'/exp OR 'randomized controlled trial (topic)'/exp) NOT ([medline]/lim OR [pubmed-not-medline]/lim) |
| Scopus | ( TITLE-ABS-KEY ( cyclooxygenase 2 inhibitor OR COX 2 inhibitor OR COX 2 specific inhibitor OR COX 2 specific inhibitors OR COX-2 inhibitor OR COX-2 specific inhibitor OR COX-2 specific inhibitors OR **cox** inhibitor OR **cox** specific inhibitor OR **coxibs** OR coxibs OR cyclooxygenase 2 inhibitors OR rofecoxib OR celecoxib OR valdecoxib OR parecoxib OR etoricoxib OR lumiracoxib ) ) AND ( ALL ( kidney OR renal OR tolerability OR safety ) ) AND NOT ( INDEX ( medline ) OR INDEX ( embase ) ) |
| Central | Title abstract keyword ("COX-2 inhibitor" OR coxib) AND (kidney OR renal OR tolerability OR safety) |
| Web of Science | cox 2 inhibitor OR coxib (Todos os campos) and kidney OR renal OR tolerability OR safety (Todos os campos) AND TS= clinical trial* OR TS=research design OR TS=comparative stud* OR TS=evaluation stud* OR TS=controlled trial* OR TS=follow-up stud* OR TS=prospective stud* OR TS=random* OR TS=placebo* OR TS=(single blind*) OR TS=(double blind*) |

**Supplementary Material 2:** Reasons for exclusion and the corresponding list of references of fully assessed studies (n = 25)

| Did not assess renal effects (n = 6) |
| --- |
| 1. Kamali K, Nikbakht J, Ayubi E, Nabizadeh M, Sarhadi S. Comparison of the Efficacy of Oxybutynin, Phenazopyridine, Celecoxib, and Placebo in the Treatment of Urinary Tract Symptoms after BCG Therapy in Patients with Bladder Tumors. Urol J. 2020;18(4):439-44. |
| 2. Karamanlioğlu B, Arar C, Alagöl A, Colak A, Gemlik I, Süt N. Preoperative oral celecoxib versus preoperative oral rofecoxib for pain relief after thyroid surgery. Eur J Anaesthesiol. 2003;20(6):490-5. |
| 3. Kivitz AJ, Espinoza LR, Sherrer YR, Liu-Dumaw M, West CR. A comparison of the efficacy and safety of celecoxib 200 mg and celecoxib 400 mg once daily in treating the signs and symptoms of psoriatic arthritis. Semin Arthritis Rheum. 2007;37(3):164-73. |
| 4. Korn S, Vassil TC, Kotey PN, Fricke JR, Jr. Comparison of rofecoxib and oxycodone plus acetaminophen in the treatment of acute pain: a randomized, double-blind, placebo-controlled study in patients with moderate to severe postoperative pain in the third molar extraction model. Clin Ther. 2004;26(5):769-78. |
| 5. Smith MR, Manola J, Kaufman DS, Oh WK, Bubley GJ, Kantoff PW. Celecoxib versus placebo for men with prostate cancer and a rising serum prostate-specific antigen after radical prostatectomy and/or radiation therapy. J Clin Oncol. 2006;24(18):2723-8. |
| 6. Soininen H, West C, Robbins J, Niculescu L. Long-term efficacy and safety of celecoxib in Alzheimer's disease. Dement Geriatr Cogn Disord. 2007;23(1):8-21. |
| Did not assess the intervention (n = 8) |
| 7. Ehrich EW, Schnitzer TJ, McIlwain H, Levy R, Wolfe F, Weisman M, et al. Effect of specific COX-2 inhibition in osteoarthritis of the knee: a 6 week double blind, placebo controlled pilot study of rofecoxib. Rofecoxib Osteoarthritis Pilot Study Group. J Rheumatol. 1999;26(11):2438-47. |
| 8. Geusens PP, Truitt K, Sfikakis P, Zhao PL, DeTora L, Shingo S, et al. A placebo and active comparator-controlled trial of rofecoxib for the treatment of rheumatoid arthritis. Scand J Rheumatol. 2002;31(4):230-8. |
| 9. Hawkey CJ, Laine L, Simon T, Quan H, Shingo S, Evans J. Incidence of gastroduodenal ulcers in patients with rheumatoid arthritis after 12 weeks of rofecoxib, naproxen, or placebo: a multicentre, randomised, double blind study. Gut. 2003;52(6):820-6. |
| 10. Katz N, Ju WD, Krupa DA, Sperling RS, Bozalis Rodgers D, Gertz BJ, et al. Efficacy and safety of rofecoxib in patients with chronic low back pain: results from two 4-week, randomized, placebo-controlled, parallel-group, double-blind trials. Spine (Phila Pa 1976). 2003;28(9):851-8; discussion 9. |
| 11. Kivitz AJ, Greenwald MW, Cohen SB, Polis AB, Najarian DK, Dixon ME, et al. Efficacy and safety of rofecoxib 12.5 mg versus nabumetone 1,000 mg in patients with osteoarthritis of the knee: a randomized controlled trial. J Am Geriatr Soc. 2004;52(5):666-74. |
| 12. Schnitzer TJ, Truitt K, Fleischmann R, Dalgin P, Block J, Zeng Q, et al. The safety profile, tolerability, and effective dose range of rofecoxib in the treatment of rheumatoid arthritis. Phase II Rofecoxib Rheumatoid Arthritis Study Group. Clin Ther. 1999;21(10):1688-702. |
| 13. Truitt KE, Sperling RS, Ettinger WH, Jr., Greenwald M, DeTora L, Zeng Q, et al. A multicenter, randomized, controlled trial to evaluate the safety profile, tolerability, and efficacy of rofecoxib in advanced elderly patients with osteoarthritis. Aging (Milano). 2001;13(2):112-21. |
| 14. van Adelsberg J, Gann P, Ko AT, Damber JE, Logothetis C, Marberger M, et al. The VIOXX in Prostate Cancer Prevention study: cardiovascular events observed in the rofecoxib 25 mg and placebo treatment groups. Curr Med Res Opin. 2007;23(9):2063-70. |
| Not a randomized controlled trial (n = 9) |
| 15. Arber N, Spicak J, Rácz I, Zavoral M, Breazna A, Gerletti P, et al. Five-year analysis of the prevention of colorectal sporadic adenomatous polyps trial. Am J Gastroenterol. 2011;106(6):1135-46. |
| 16. Bensen WG, Zhao SZ, Burke TA, Zabinski RA, Makuch RW, Maurath CJ, et al. Upper gastrointestinal tolerability of celecoxib, a COX-2 specific inhibitor, compared to naproxen and placebo. J Rheumatol. 2000;27(8):1876-83. |
| 17. Bertagnolli MM, Eagle CJ, Zauber AG, Redston M, Breazna A, Kim K, et al. Five-year efficacy and safety analysis of the Adenoma Prevention with Celecoxib Trial. Cancer Prev Res (Phila). 2009;2(4):310-21. |
| 18. Boswell DJ, Ostergaard K, Philipson RS, Hodge RA, Blum D, Brown JC, et al. Evaluation of GW406381 for treatment of osteoarthritis of the knee: two randomized, controlled studies. Medscape J Med. 2008;10(11):259. |
| 19. Chan AT, Hsu M, Zauber AG, Hawk ET, Bertagnolli MM. The influence of UGT1A6 variants and aspirin use in a randomized trial of celecoxib for prevention of colorectal adenoma. Cancer Prev Res (Phila). 2012;5(1):61-72. |
| 20. Curtis SP, Bockow B, Fisher C, Olaleye J, Compton A, Ko AT, et al. Etoricoxib in the treatment of osteoarthritis over 52-weeks: a double-blind, active-comparator controlled trial [NCT00242489]. BMC Musculoskelet Disord. 2005;6:58. |
| 21. Daniels S, Gitton X, Zhou W, Stricker K, Barton S. Efficacy and tolerability of lumiracoxib 200 mg once daily for treatment of primary dysmenorrhea: results from two randomized controlled trials. J Womens Health (Larchmt). 2008;17(3):423-37. |
| 22. Nussmeier NA, Whelton AA, Brown MT, Langford RM, Hoeft A, Parlow JL, et al. Complications of the COX-2 inhibitors parecoxib and valdecoxib after cardiac surgery. N Engl J Med. 2005;352(11):1081-91. |
| 23. Sheldon EA, Beaulieu A, Paster Z, Yu S, Rebuli R. Long-term efficacy and safety of lumiracoxib 100 mg: an open-label extension of a 13-week randomized controlled trial in patients with primary osteoarthritis of the knee. Clin Exp Rheumatol. 2008;26(4):611-9. |
| Full text unavailable (n = 2) |
| 24. Hegazy EM, El-Hady NA, Abdallah MW. Comparative study of postoperative analgesic effects of parecoxib versus ketorolac and placebo in cervical disc surgery. Egyptian Journal of Anaesthesia. 2003;19(2):179-82. |
| 25. Krammer G, Stricker K, Jayawardene S, Rosemary R. Evaluation of the analgesic efficacy of two lumiracoxib regimens in the treatment of post-operative dental pain: Single-centre, double-blind, randomised placebo-controlled, parallel-group study. Journal of Clinical Research. 2008;11(25-39):25-39. |

**Supplementary Material 3:** Characteristics of the studies included using our systematic review process

| Study | Study dates | Region | Clinical condition | Population | Coxib | Dose | N in coxib | N in placebo | Trial duration | Funding source |
| --- | --- | --- | --- | --- | --- | --- | --- | --- | --- | --- |
| Arber 2006 | 2001-2005 | America, Europe, Africa, Asia | Colorectal cancer | Men, women, elderly | Celecoxib | 400mg | 933 | 628 | 36 months | Pfizer |
| Bensen 1999 | 1996-1997 | Canada, United States | Osteoarthritis | Men, women elderly | Celecoxib | 50mg | 203 | 203 | 1-3 months | No information |
|  |  |  |  |  |  | 100mg | 197 |  |  |  |
|  |  |  |  |  |  | 200mg | 202 |  |  |  |
| Bertagnolli 2006 | 2000-2006 | United States, Australia, Canada, United Kingdom | Colorectal cancer | Men, women, elderly | Celecoxib | 200mg | 685 | 679 | 36 months | Pfizer and National Cancer Institute |
|  |  |  |  |  |  | 400mg | 671 |  |  |  |
| Bickham 2016 | 2010-2014 | America, Europe, Africa, Asia | Rheumatoid arthritis | Men, women elderly | Etoricoxib | 60mg | 818 | 118 | 1-3 months | Merck |
|  |  |  |  |  |  | 90mg | 468 |  |  |  |
| Bingham 2007a | 2004-2005 | United States | Osteoarthritis | Men, women | Etoricoxib | 30mg | 231 | 127 | 1-3 months | Merck |
|  |  |  |  |  | Celecoxib | 200mg | 241 |  |  |  |
| Bingham 2007 b | 2004-2005 | United States | Osteoarthritis | Men, women | Etoricoxib | 30mg | 244 | 117 | 1-3 months | Merck |
|  |  |  |  |  | Celecoxib | 200mg | 247 |  |  |  |
| Birbara 2006 | 2003-2004 | United States | Osteoarthritis | Men, women | Celecoxib | 200mg | 326 | 163 | 1-3 months | Merck |
| Camu 2002 | No information | Europe | Arthroplasty | Men, women | Valdecoxib | 20mg | 73 | 71 | 1-2 days | Pfizer and Pharmacia Corporation |
|  |  |  |  |  |  | 40mg | 73 |  |  |  |
| Cannon 2008 | 2002-2003 | United States | Osteoarthritis | Men, women | Etoricoxib | 90mg | 108 | 111 | 1-3 months | Merck |
|  |  |  |  |  | Celecoxib | 200mg | 107 |  |  |  |
| Chen 2016 | No information | No information | Endonasal surgery | Men, women | Parecoxib | 40mg | 31 | 33 | 1-2 days | No information |
| Coats 2004 | No information | Canada, United States | Pain | Men, women, elderly | Valdecoxib | 40mg | 148 | 145 | 1-3 months | No information |
| Collantes 2002 | 1999-2000 | America, Europe, Africa, Asia | Rheumatoid arthritis | Men, women | Etoricoxib | 90mg | 353 | 357 | 1-3 months | Merck, Novartis, Lilly, Roche, Aventis, Pfizer, AstraZeneca, Immunex, Isotechnika, Janssen-Ortho, Knoll and Abbott |
| Cryer 2011 a | 2008 | United States | Osteoarthritis | Men, women, elderly | Celecoxib | 200mg | 247 | 124 | 1-3 months | Pozen and AstraZeneca |
| Cryer 2011 b | 2008 | United States | Osteoarthritis | Men, women, elderly | Celecoxib | 200mg | 247 | 124 | 1-3 months | Pozen and AstraZeneca |
| Elmets 2010 | 2001-2006 | United States | Actinic keratosis | Men, women, elderly | Celecoxib | 200mg | 122 | 118 | 6-11 months | Pfizer and National Cancer Institute |
| Essex 2018 | 2002-2003 | South Korea | Arthroplasty | Men, women, elderly | Parecoxib | 40mg | 58 | 58 | 1-2 days | Pfizer |
| Fleischmann 2006 | No information | America, Europe | Osteoarthritis | Men, women, elderly | Lumiracoxib | 200mg | 465 | 232 | 1-3 months | Novartis |
|  |  |  |  |  |  | 400mg | 465 |  |  |  |
|  |  |  |  |  | Celecoxib | 200mg | 446 |  |  |  |
| Geusens 2004 | No information | Europe, Africa, Asia, Oceania | Rheumatoid arthritis | Men, women, elderly | Lumiracoxib | 200mg | 280 | 284 | 6-11 months | Novartis |
|  |  |  |  |  |  | 400mg | 281 |  |  |  |
| Ghosh 2007 | No information | India | Osteoarthritis | Men, women | Etoricoxib | 90mg | 162 | 123 | 1-3 months | No information |
| Gibofsky 2003 | No information | Canada, United States | Osteoarthritis | Men, women | Celecoxib | 200mg | 189 | 96 | 1-3 months | Pharmacia Corporation |
| Gottesdiener 2002 | 1998-1999 | United States | Osteoarthritis | Men, women, elderly | Etoricoxib | 5mg | 117 | 60 | 1-3 months | Merck |
|  |  |  |  |  |  | 10mg | 114 |  |  |  |
|  |  |  |  |  |  | 30mg | 102 |  |  |  |
|  |  |  |  |  |  | 60mg | 112 |  |  |  |
|  |  |  |  |  |  | 90mg | 112 |  |  |  |
| Greenwald 2011 | 2006-2008 | United States, Canada, Colombia, Switzerland | Rheumatoid arthritis | Men, women, elderly | Etoricoxib | 10mg | 154 | 161 | 1-3 months | Merck |
|  |  |  |  |  |  | 30mg | 151 |  |  |  |
|  |  |  |  |  |  | 60mg | 140 |  |  |  |
|  |  |  |  |  |  | 90mg | 155 |  |  |  |
| Grifka 2004 | No information | Germany, Canada, France, Italy | Osteoarthritis | Men, women | Lumiracoxib | 200mg | 205 | 196 | 1-3 months | Novartis |
|  |  |  |  |  |  | 400mg | 193 |  |  |  |
| Hubbard 2003 | 1999-2000 | Belgium, France, Germany, Sweden | Arthroplasty | Men, women, elderly | Parecoxib | 20mg | 65 | 63 | 1-2 days | Pharmacia Corporation |
|  |  |  |  |  |  | 40mg | 67 |  |  |  |
| Ishiguro 2015 | 2010 | Japan | Pain | Men, women, elderly | Celecoxib | 400mg | 248 | 124 | 1-2 days | Astellas Pharma |
| Kivitz 2001 | No information | Canada, United States | Osteoarthritis | Men, women, elderly | Celecoxib | 100mg | 216 | 218 | 1-3 months | Pfizer and Pharmacia Corporation |
|  |  |  |  |  |  | 200mg | 207 |  |  |  |
|  |  |  |  |  |  | 400mg | 213 |  |  |  |
| Lee 2017 | 2013 | South Korea | Osteoarthritis | Men, women, elderly | Celecoxib | 200mg | 145 | 71 | 1-3 months | No information |
| Lehmann 2005 | No information | No information | Osteoarthritis | Men, women, elderly | Lumiracoxib | 100mg | 840 | 424 | 1-3 months | Novartis |
|  |  |  |  |  | Celecoxib | 200mg | 420 |  |  |  |
| Leung 2002 | No information | America, Europe, Africa, Asia, Oceania | Osteoarthritis | Men, women, elderly | Etoricoxib | 60mg | 224 | 56 | 1-3 months | Merck |
| Mahagna 2016 | 2008-2012 | Israel | Fibromyalgia | Women, elderly | Etoricoxib | 90mg | 32 | 32 | 1-3 months | MSD |
| Matsumoto 2002 | No information | United States | Rheumatoid arthritis | Men, women, elderly | Etoricoxib | 90mg | 323 | 323 | 1-3 months | Merck |
| McKenna 2001 | No information | United States | Osteoarthritis | Men, women, elderly | Celecoxib | 100mg | 201 | 200 | 1-3 months | Pharmacia Corporation |
| Pallay 2004 | No information | United States | Pain | Men, women, elderly | Etoricoxib | 90mg | 109 | 110 | 1-3 months | Merck |
|  |  |  |  |  |  | 60mg | 106 |  |  |  |
| Papadimitrakopoulou 2008 | 2000-2004 | United States | Oral premalignant lesions | Men, women, elderly | Celecoxib | 100mg | 17 | 18 | 1-3 months | Pfizer |
|  |  |  |  |  |  | 200mg | 15 |  |  |  |
| Puopolo 2007 | No information | United States, Latin America | Osteoarthritis | Men, women, elderly | Etoricoxib | 30mg | 224 | 111 | 1-3 months | Merck |
| Rasmussen 2005 | No information | United States | Pain | Men, women, elderly | Etoricoxib | 120mg | 80 | 75 | 1-2 weeks | Merck |
| Reginster 2007 | No information | United States, Canada, Australia, Europe | Osteoarthritis | Men, women, elderly | Etoricoxib | 60mg | 446 | 112 | 1-3 months | No information |
| Schnitzer 2004 | 1999 | North America, Europe | Osteoarthritis | Men, women, elderly | Lumiracoxib | 50mg | 98 | 97 | 1-3 months | No information |
|  |  |  |  |  |  | 100mg | 96 |  |  |  |
|  |  |  |  |  |  | 200mg | 99 |  |  |  |
|  |  |  |  |  |  | 400mg | 99 |  |  |  |
| Schnitzer 2005 | No information | No information | Rheumatoid arthritis | Men, women, elderly | Lumiracoxib | 50mg | 102 | 99 | 1-3 months | Novartis |
|  |  |  |  |  |  | 100mg | 97 |  |  |  |
|  |  |  |  |  |  | 200mg | 93 |  |  |  |
|  |  |  |  |  |  | 400mg | 87 |  |  |  |
| Schnitzer 2011 | 2005-2012 | Canada, United States, Germany, Italy, United Kingdom | Osteoarthritis | Men, women, elderly | Lumiracoxib | 100mg | 427 | 416 | 1-3 months | Novartis |
|  |  |  |  |  | Celecoxib | 200mg | 419 |  |  |  |
| Schwartz 2002 | No information | No information | Healthy elderly | Elderly | Celecoxib | 200mg | 17 | 16 | 1-2 weeks | Merck |
| Simon 1999 | 1996-1998 | Canada, United States | Rheumatoid arthritis | Men, women, elderly | Celecoxib | 100mg | 240 | 231 | 1-3 months | G.D. Searle & Co |
|  |  |  |  |  |  | 200mg | 235 |  |  |  |
|  |  |  |  |  |  | 400mg | 218 |  |  |  |
| Smugar 2006 a | 1999-2000 | United States | Osteoarthritis | Men, women, elderly | Celecoxib | 200mg | 456 | 150 | 1-3 months | Merck |
| Smugar 2006 b | 1999-2000 | United States | Osteoarthritis | Men, women, elderly | Celecoxib | 200mg | 460 | 151 | 1-3 months | Merck |
| Stoltz 2002 | No information | No information | Healthy elderly | Elderly | Parecoxib | 40mg | 31 | 32 | 1-2 weeks | Pharmacia Corporation |
| Tannenbaum 2004 | No information | Europe | Osteoarthritis | Men, women, elderly | Lumiracoxib | 200mg | 487 | 243 | 1-3 months | Novartis |
|  |  |  |  |  |  | 400mg | 491 |  |  |  |
|  |  |  |  |  | Celecoxib | 200mg | 481 |  |  |  |
| Tsoukas 2006 | No information | United States | Hemophilic arthropathy | Men, women, elderly, teenagers | Etoricoxib | 90mg | 51 | 51 | 1-3 months | Merck |
| Wiesenhutter 2005 | 2003 | United States | Osteoarthritis | Men, women, elderly | Etoricoxib | 30mg | 214 | 104 | 1-3 months | Merck |
| Williams 2000 | No information | United States | Osteoarthritis | Men, women, elderly | Celecoxib | 100mg | 231 | 232 | 1-3 months | No information |
|  |  |  |  |  |  | 200mg | 223 |  |  |  |

**Supplementary Material 4:** Meta-regression for the relative risk (RR) of hypertension according to the decade the study was conducted


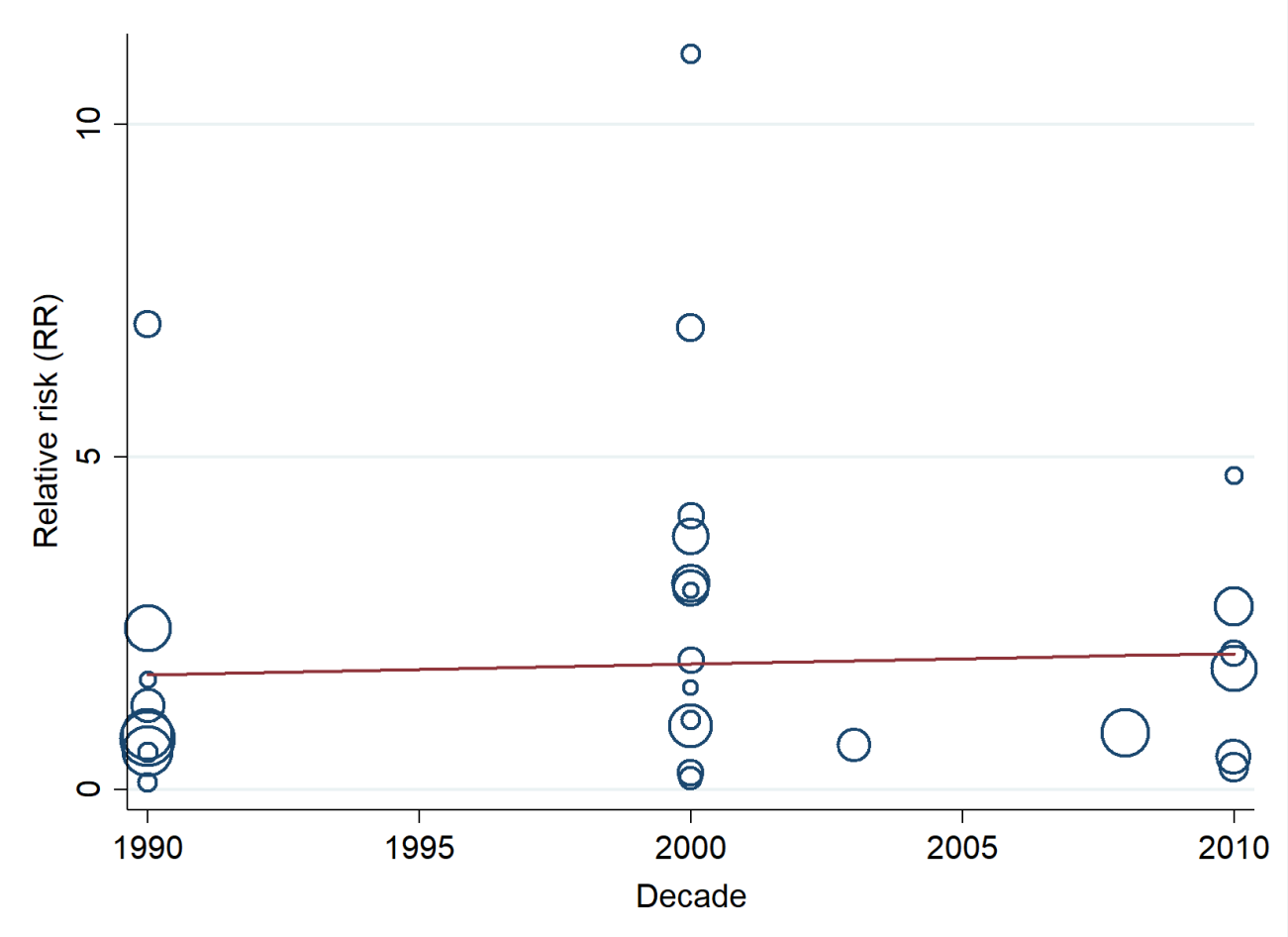


**Supplementary Material 5:** Meta-regression for the relative risk (RR) of hypertension according to the participants’ age


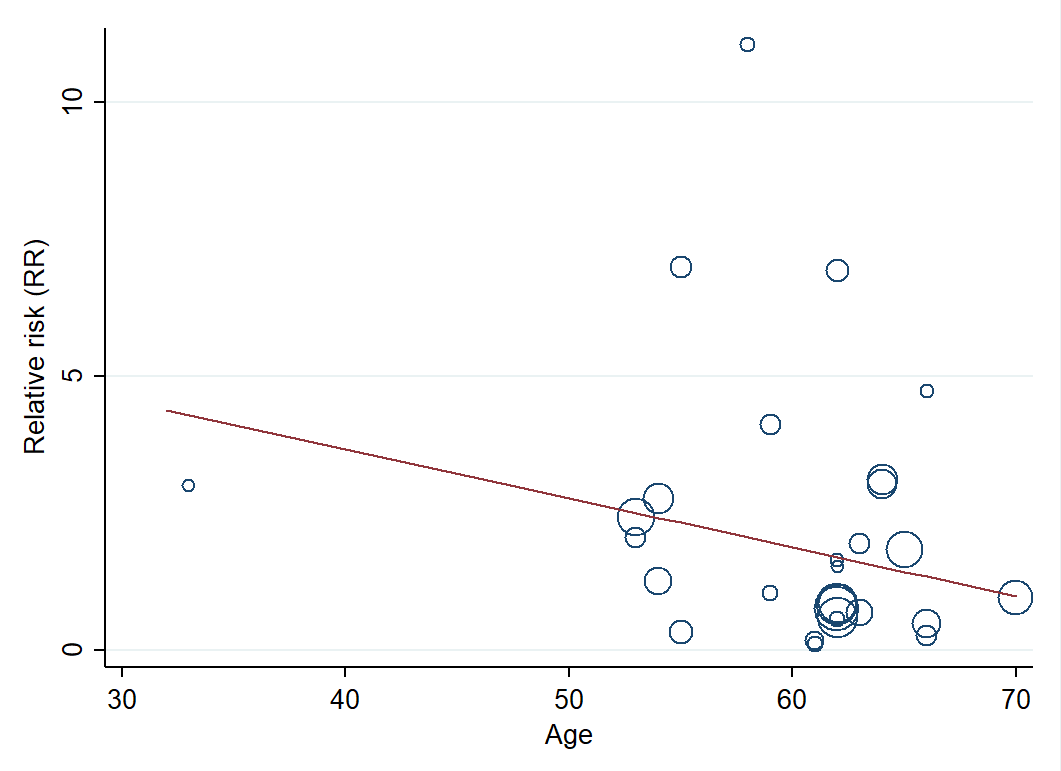


**Supplementary Material 6:** Meta-regression for the relative risk (RR) of hypertension according to the risk of bias of the studies


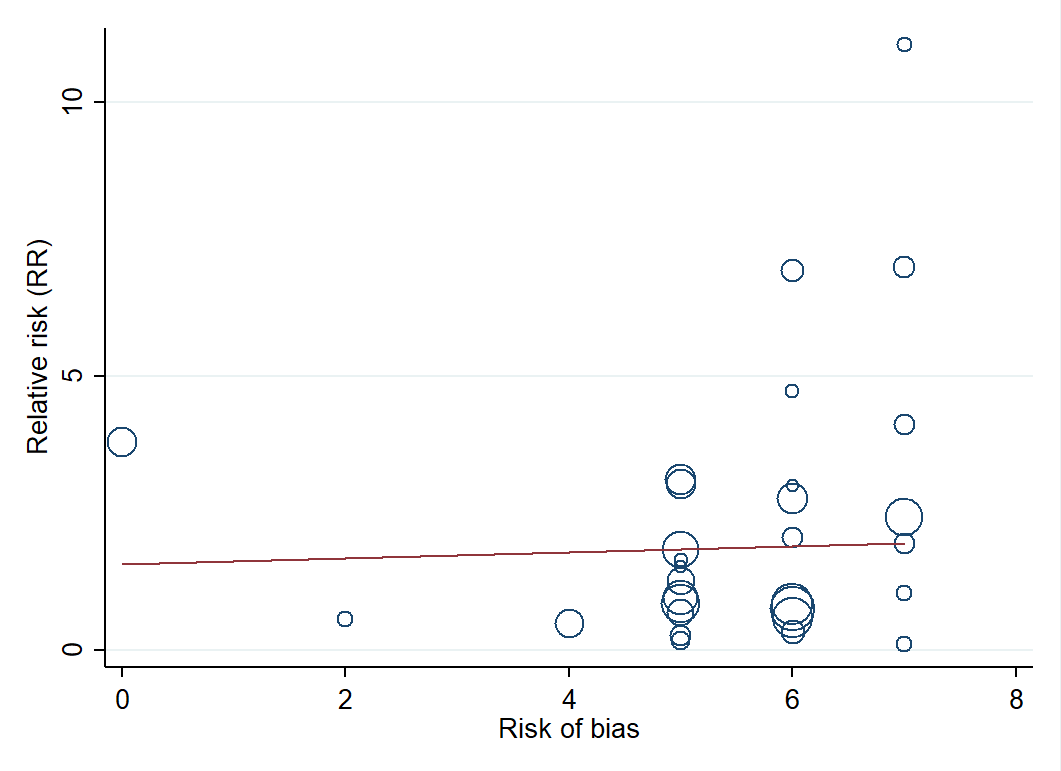


**Supplementary Material 7:** Relative risk (RR) of edema of selective cyclooxygenase-2 inhibitor anti-inflammatory drugs compared to placebo according to the study size (standard error [SE] of the RR)


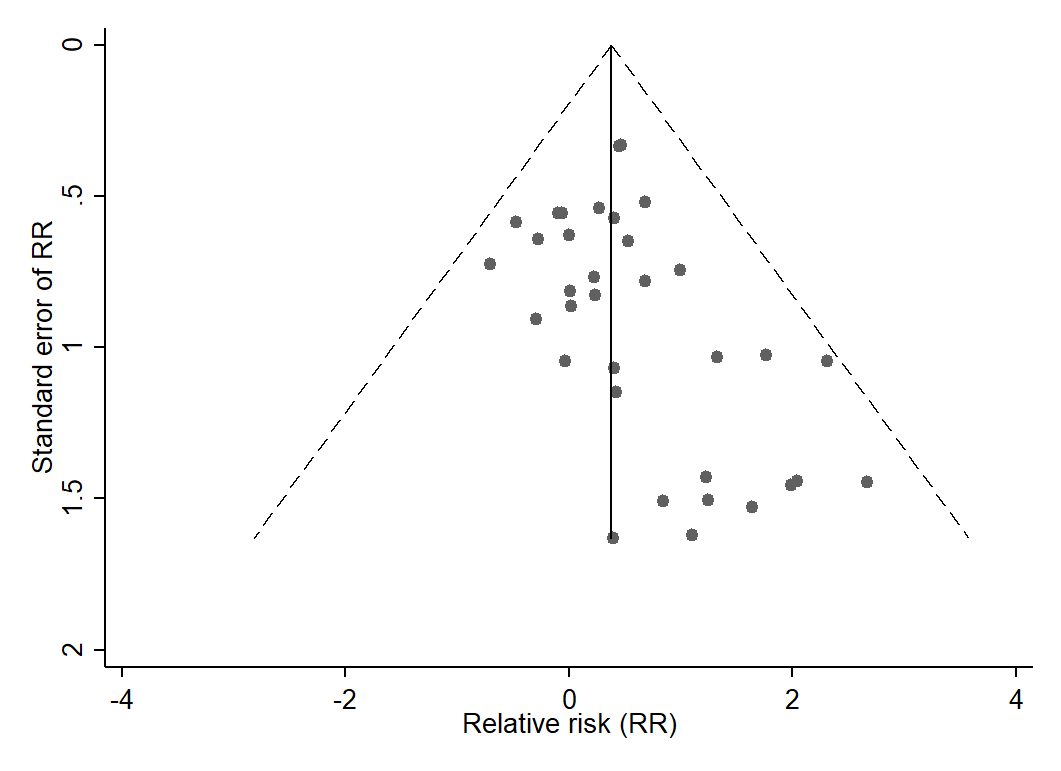


**Supplementary Material 8:** Relative risk (RR) of hypertension of selective cyclooxygenase-2 inhibitor anti-inflammatory drugs compared to placebo according to study size (standard error [SE] of the RR)

**
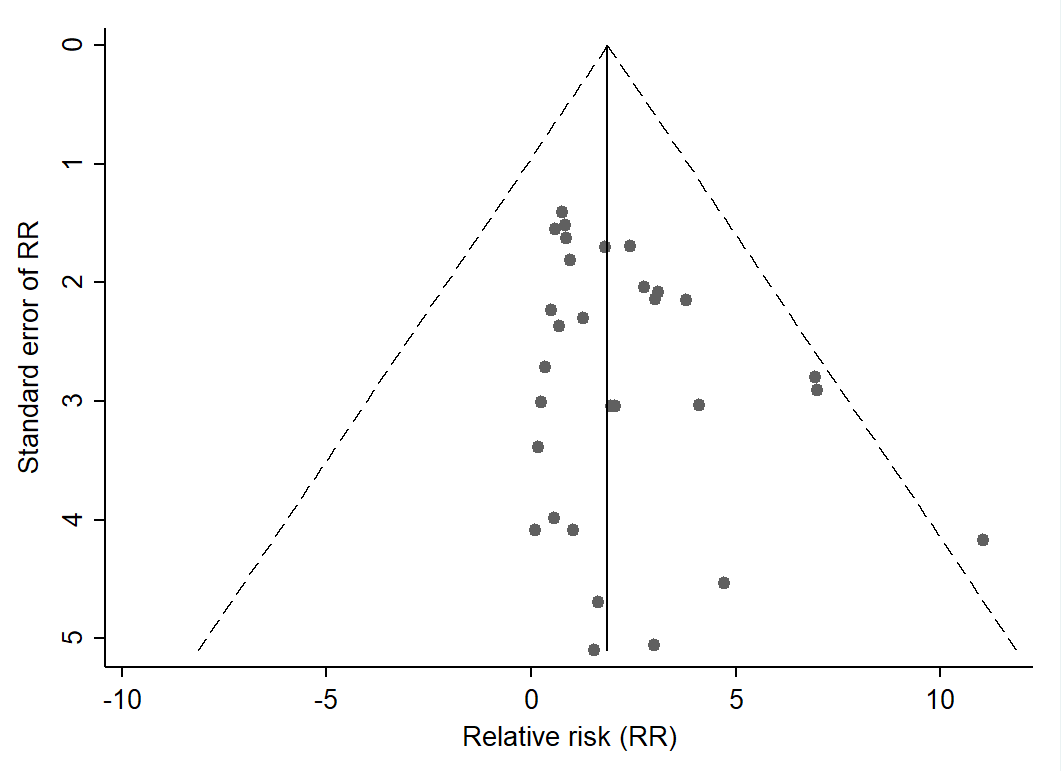
**

**Supplementary Material 9:** Summary of findings and certainty of evidence of the renal effects of selective cyclooxygenase-2 inhibitor anti-inflammatory drugs compared to placebo, in relative risk (RR) and 95% confidence interval (95%CI)

| Outcomes | N° of participants (studies) | Certainty of the evidence | RR (95%CI) | Anticipated absolute effects | |
| --- | --- | --- | --- | --- | --- |
|  |  |  |  | Risk in placebo | Risk difference with coxib |
| Critical outcomes | | | | | |
| Elevated creatinine | 3195 (4) | ⨁⨁◯◯ Low^a,b,c^ | 0.73 (0.21, 2.55) | 11 per 1000 | 3 fewer per 1000 (9 fewer to 17 more) |
| Renal or hypertensive outcomes (celecoxib) | 3589 (2) | ⨁⨁⨁◯ Moderate^d^ | 1.24 (1.08, 1.43) | 173 per 1000 | 42 more per 1000 (12 more to 75 more) |
| Renal and urinary disorders (celecoxib) | 4244 (3) | ⨁⨁◯◯ Low^b,d^ | 1.18 (0.65, 2.15) | 11 per 1000 | 2 more per 1000 (4 fewer to 12 more) |
| Elevated blood urea nitrogen (BUN) | 1282 (3) | ⨁⨁⨁◯ Moderate^b,c^ | 2.21 (0.35, 13.95) | 0 per 1000 | 0 fewer per 1000 (0 fewer to 0 fewer) |
| Hypertension | 16,173 (30) | ⨁⨁⨁◯ Moderate^b^ | 1.26 (0.91, 1.76) | 17 per 1000 | 5 more per 1000 (2 fewer to 13 more) |
| Hypertension (celecoxib) | 5755 (11) | ⨁⨁⨁◯ Moderate^b^ | 0.94 (0.58, 1.55) | 15 per 1000 | 1 fewer per 1000 (6 fewer to 8 more) |
| Hypertension (etoricoxib) | 6560 (13) | ⨁⨁⨁◯ Moderate^e^ | 1.98 (1.14, 3.46) | 15 per 1000 | 15 more per 1000 (2 more to 37 more) |
| Hypertension (valdecoxib) | 217 (1) | ⨁⨁◯◯ Low^b,e^ | 0.49 (0.10, 2.35) | 42 per 1000 | 22 fewer per 1000 (38 fewer to 57 more) |
| Hypertension (parecoxib) | 311 (2) | ⨁⨁⨁◯ Moderate^b^ | 0.67 (0.21, 2.16) | 66 per 1000 | 22 fewer per 1000 (52 fewer to 77 more) |
| Hypertension (lumiracoxib) | 3330 (3) | ⨁⨁⨁◯ Moderate^b^ | 1.20 (0.50, 2.86) | 19 per 1000 | 4 more per 1000 (9 fewer to 35 more) |
| Important outcomes |  |  |  |  |  |
| Edema | 19,754 (34) | ⨁⨁⨁◯ Moderate^f^ | 1.46 (1.15, 1.86) | 16 per 1000 | 7 more per 1000 (2 more to 14 more) |
| Edema (celecoxib) | 8413 (14) | ⨁⨁⨁◯ Moderate^f^ | 1.52 (1.04, 2.24) | 13 per 1000 | 7 more per 1000 (0 fewer to 16 more) |
| Edema (etoricoxib) | 6496 (13) | ⨁⨁⨁◯ Moderate^b^ | 1.52 (0.95, 2.43) | 14 per 1000 | 7 more per 1000 (1 fewer to 19 more) |
| Edema (lumiracoxib) | 4782 (6) | ⨁⨁◯◯ Low^b,f^ | 1.31 (0.87, 1.98) | 27 per 1000 | 8 more per 1000 (3 fewer to 26 more) |
| Edema (parecoxib) | 63 (1) | ⨁⨁⨁◯ Moderate^b^ | 5.16 (0.26, 103.27) | 0 per 1000 | 0 fewer per 1000 (0 fewer to 0 fewer) |
| Glycosuria (celecoxib) | 372 (1) | ⨁⨁⨁◯ Moderate^b,c^ | 0.40 (0.11, 1.46) | 40 per 1000 | 24 fewer per 1000 (36 fewer to 19 more) |
| Hematuria | 1133 (2) | ⨁⨁⨁◯ Moderate^b,c^ | 1.81 (0.60, 5.43) | 14 per 1000 | 11 more per 1000 (6 fewer to 62 more) |
| Not important outcomes |  |  |  |  |  |
| Urinary retention | 487 (3) | ⨁◯◯◯ Very low^b,c,f,g^ | 0.96 (0.13, 7.15) | 36 per 1000 | 0 fewer per 1000 (31 fewer to 235 more) |
| Urinary tract infection | 6587 (10) | ⨁⨁◯◯ Low^b,c,h^ | 0.81 (0.56, 1.16) | 27 per 1000 | 8 fewer per 1000 (15 fewer to 4 more) |
| Urinary tract infection (celecoxib) | 2604 (3) | ⨁◯◯◯ Very low^b,g,h^ | 0.78 (0.39, 1.57) | 27 per 1000 | 16 fewer per 1000 (24 fewer to 23 more) |
| Urinary tract infection (etoricoxib) | 1657 (4) | ⨁⨁◯◯ Low^b,h^ | 1.10 (0.44, 2.73) | 33 per 1000 | 3 more per 1000 (18 fewer to 57 more) |
| Urinary tract infection (lumiracoxib) | 2109 (2) | ⨁◯◯◯ Very low^b,f,h^ | 0.71 (0.36, 1.37) | 21 per 1000 | 6 fewer per 1000 (14 fewer to 8 more) |
| Urinary tract infection (valdecoxib) | 217 (1) | ⨁◯◯◯ Very low^b,e,f,h^ | 0.49 (0.10, 2.35) | 42 per 1000 | 22 fewer per 1000 (38 fewer to 57 more) |
| Discontinuation due to elevated creatinine | 1221 (1) | ⨁⨁⨁◯ Moderate^b^ | 0.75 (0.03, 18.30) | 0 per 1000 | 0 fewer per 1000 (0 fewer to 0 fewer) |
| Discontinuation due to hypertension | 5769 (10) | ⨁⨁◯◯ Low^b,e^ | 0.99 (0.46, 2.15) | 3 per 1000 | 0 fewer per 1000 (2 fewer to 3 more) |
| Discontinuation due to hypertension (celecoxib) | 1462 (3) | ⨁⨁◯◯ Low^b,e^ | 1.04 (0.19, 5.82) | 1 per 1000 | 0 fewer per 1000 (1 fewer to 7 more) |
| Discontinuation due to hypertension (etoricoxib) | 4307 (7) | ⨁⨁◯◯ Low^b,e^ | 0.98 (0.41, 2.33) | 4 per 1000 | 0 fewer per 1000 (2 fewer to 5 more) |
| Discontinuation due to edema | 7109 (13) | ⨁⨁⨁◯ Moderate^b^ | 1.75 (0.77, 3.98) | 0 per 1000 | 0 fewer per 1000 (0 fewer to 1 more) |
| Discontinuation due to edema (celecoxib) | 2073 (4) | ⨁⨁◯◯ Low^b,e^ | 2.47 (0.64, 9.55) | 1 per 1000 | 0 fewer per 1000 (1 fewer to 5 more) |
| Discontinuation due to edema (etoricoxib) | 5036 (9) | ⨁⨁◯◯ Low^b,e^ | 1.43 (0.51, 4.03) | 0 per 1000 | 0 fewer per 1000 (0 fewer to 0 fewer) |

^a^High risk for incomplete outcome data.

^b^Confidence interval not significant.

^c^Many studies reporting this outcome used a threshold to report adverse reactions.

^d^Most studies were stopped earlier than planned.

^e^High proportion of studies with poor randomization and allocation concealment.

^f^Most studies were not blinded for outcome assessment.

^g^Moderate heterogeneity.

^h^The presence of leukocytes was considered suggestive of urinary infection.
